# Supplementary figures and images for: Impact of the Technical Snow Production Process on Bacterial Community Composition, Antibacterial Resistance Genes, and Antibiotic Input—A Dual Effect of the Inevitable
Source: Int J Mol Sci. 2025 Mar 19;26(6):2771. doi: 10.3390/ijms26062771 (PMC11942910; doi:10.3390/ijms26062771)

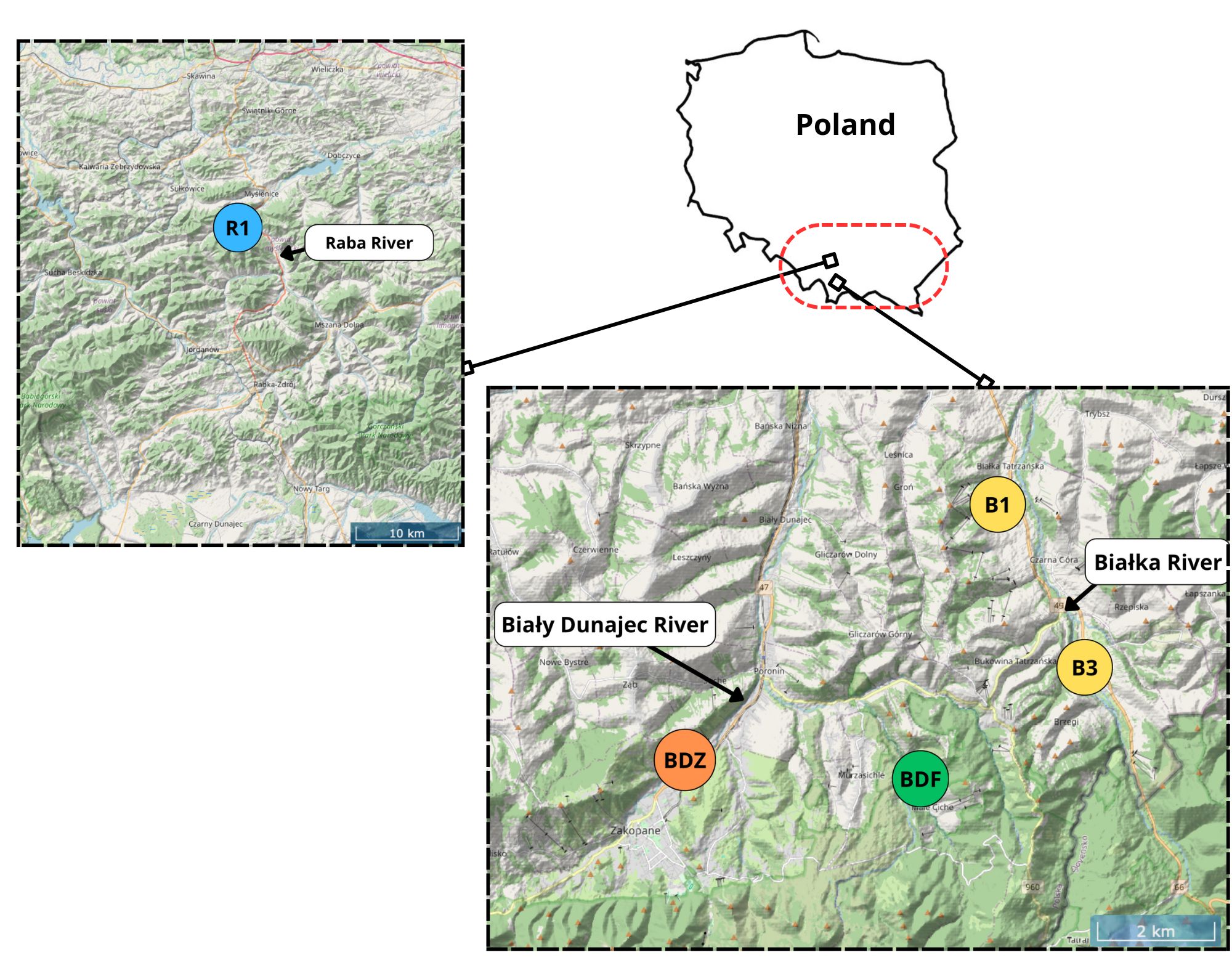

Supplement: Supplementary file 1 [file ijms-26-02771-s001.zip › Supplementary materials/Supplementary Figure_S1_study sites.jpg]
